# Functional Investigation of Iron-Responsive Microsomal Proteins, including MirC, in *Aspergillus fumigatus*

**DOI:** 10.3389/fmicb.2017.00418

**Published:** 2017-03-17

**Authors:** Eoin D. Mulvihill, Nicola M. Moloney, Rebecca A. Owens, Stephen K. Dolan, Lauren Russell, Sean Doyle

**Affiliations:** ^1^Department of Biology, Maynooth UniversityKildare, Ireland; ^2^Department of Biochemistry, University of CambridgeCambridge, UK

**Keywords:** siderophore, fungal proteomics, NRPS, membrane proteins, oxidative stress

## Abstract

The functionality of many microsome-associated proteins which exhibit altered abundance in response to iron limitation in *Aspergillus fumigatus* is unknown. Here, we generate and characterize eight gene deletion strains, and of most significance reveal that MirC (AFUA_2G05730) contributes to the maintenance of intracellular siderophore [ferricrocin (FC)] levels, augments conidiation, confers protection against oxidative stress, exhibits an intracellular localization and contributes to fungal virulence in the *Galleria mellonella* animal model system. FC levels were unaffected following deletion of all other genes encoding microsome-associated proteins. MirC does not appear to play a role in either siderophore export from, or uptake into, *A. fumigatus*. Label-free quantitative proteomic analysis unexpectedly revealed increased abundance of siderophore biosynthetic enzymes. In addition, increased expression of *hapX* (7.2 and 13.8-fold at 48 and 72 h, respectively; *p* < 0.001) was observed in Δ*mirC* compared to wild-type under iron-replete conditions by qRT-PCR. This was complemented by significantly elevated extracellular triacetylfusarinine C (TAFC; *p* < 0.01) and fusarinine C (FSC; *p* < 0.05) siderophore secretion. We conclude that MirC plays an important role in FC biosynthesis and contributes to the maintenance of iron homeostasis in *A. fumigatus*.

## Introduction

Many iron-requiring fungi utilize high affinity iron acquisition strategies to procure essential iron. However, an ability to exist in two oxidative states; ferric (Fe^3+^) and ferrous (Fe^2+^), means that iron can drive the generation of deleterious reactive oxygen species (Halliwell and Gutteridge, 1984). Paradoxically, iron is also required as a co-factor for antioxidant enzymes, such as peroxidases and catalases, in the form of heme. Thus, processes of oxidative stress tolerance and iron regulation are tightly interwoven to maintain cellular homeostasis (Brandon et al., 2015). In fungi, careful management of the utilization of high affinity iron uptake mechanisms is an important facet of iron metabolism. Iron sensitive transcription factors, SreA and HapX are core players underlying the global “omic” shift observed when *Aspergillus* species face iron starvation (Oberegger et al., 2001; Schrettl et al., 2008, 2010). SreA is a GATA-type transcription factor that represses high affinity iron acquisition pathways during iron sufficiency. Conversely, HapX is a bZip transcription factor that represses iron-consuming pathways during iron starvation. One of the most notable responses to iron starvation in fungi is the production of siderophores. Siderophores are low molecular mass compounds capable of solubilising ferric iron. They are secreted in the desferri-form and following external iron chelation are taken back up for intracellular storage or direct use in metabolism (Haas, 2014).

*Aspergillus fumigatus*, a filamentous fungus which produces ubiquitous airborne conidia, is the predominant causative agent of invasive aspergillosis (IA); an infection mainly affecting immune-deficient hosts with a notoriously high mortality/morbidity rate (Dagenais and Keller, 2009). *A. fumigatus* produces four siderophores; two intracellular siderophores and two extracellular siderophores. Intracellular siderophores include ferricrocin (FC) that governs iron storage and distribution in hyphae and hydroxyferricrocin (HFC) that does so in conidia. In contrast, extracellular siderophores, fusarinine C (FSC) and its acetylated derivative, triacetylfusarinine C (TAFC) are secreted from hyphae for iron uptake (Schrettl et al., 2007). The siderophore biosynthetic pathway begins with the hydroxylation of ornithine by L-ornithine *N*^5^-oxygenase, SidA to generate *N*^5^-hydroxy-L-ornithine (Schrettl et al., 2004). Following this step, the pathway splits for the production of extracellular and intracellular siderophores. The ergosterol biosynthetic intermediate, mevalonate is directed into extracellular siderophore biosynthesis following enzymatic modification with mevalonyl-CoA ligase, SidI and mevalonyl-CoA hydratase, SidH to generate anhydromevalonyl (Yasmin et al., 2012). This process occurs in peroxisomes where transacylase SidF then mediates the addition of anhydromevalonyl to *N*^5^-hydroxy-L-ornithine (Gründlinger et al., 2013b). This *N*^5^-anhydromevalonyl-*N*^5^-hydroxy-L-ornithine moiety serves as a substrate for non-ribosomal peptide synthetase (NRPS), SidD which directs the generation of FSC consisting of 3 *N*^5^-anhydromevalonyl-*N*^5^-hydroxy-L-ornithine groups linked by ester bonds. TAFC is then derived from SidG-mediated *N*^2^ acetylation of FSC (Schrettl et al., 2007). During intracellular siderophore biosynthesis, the *N*^5^-hydroxy-L-ornithine is instead acetylated by SidL and another unknown transacylase to generate *N*^5^-acetyl-*N*^5^-hydroxy-L-ornithine (Blatzer et al., 2011). This moiety then serves as the substrate for NRPS, SidC along with glycine and serine to generate FC. FC is then hydroxylated by an unknown hydroxylase to generate HFC (Schrettl et al., 2007). *A. fumigatus* secretes desferri-FSC/TAFC (FSC^−Fe^/TAFC^−Fe^) through an unknown mechanism. FSC^−Fe^/TAFC^−Fe^ chelate Fe^3+^ and the resulting siderophore:iron complexes are taken back up by siderophore-iron transporters (SITs) (Haas et al., 2003; Philpott and Protchenko, 2008). FSC^+Fe^/TAFC^+Fe^ are then cytosolically hydrolysed by esterases and the released iron is used by metabolic machinery, stored in vacuoles or ferri-FC (FC^+Fe^) (Kragl et al., 2007; Gründlinger et al., 2013a).

The ability to synthesize and utilize siderophores constitutes an important part of the pathogen weaponry at the interface with the host (Cassat and Skaar, 2013). Importantly, using a SidA deletion mutant, siderophore mediated iron acquisition was shown to be essential for virulence of *A. fumigatus* in mice specifically indicating siderophore production as a plausible therapeutic target for this potentially devastating infection (Schrettl et al., 2004; Hissen et al., 2005). Targeting siderophore-mediated iron acquisition has been proposed as a therapeutic strategy against microbial infection via “Trojan Horse” drug delivery or interference with siderophore biosynthesis (Miethke and Marahiel, 2007; Lamb, 2015). Moreover, siderophore transporters represent one of the few protein families unique to fungi and thus, represent promising antifungal targets.

SITs are a fungal-specific protein family within the major facilitator superfamily (MFS) (Pao et al., 1998; Saier et al., 1999). Interestingly, SITs are also conserved in fungi incapable of producing siderophores (Philpott and Protchenko, 2008). A *Saccharomyces cerevisiae* strain deficient in high affinity iron acquisition was used to determine the substrate specificity of SITs from *Aspergillus nidulans*. Using this approach, MirA was found to be specific for enterobactin and MirB for TAFC^+Fe^ (Haas et al., 2003). The *A. fumigatus* genome encodes seven putative SITs (Haas et al., 2008), which include five transporters induced under iron limitation (Moloney et al., 2016); Sit1, AFUA_3G03670, MirA/D, MirB, and MirC. Though MirA and MirB have been implicated in siderophore transport in *A. nidulans*, a role for MirC has yet to be demonstrated (Haas et al., 2003).

Significant recent advances including extensive transcriptional and proteomic analysis, has yielded insight into the regulation and functional remodeling undertaken during iron acquisition in *A. fumigatus*. However, the location and mechanisms underlying the membrane transport of siderophores remain to be fully elucidated in this important pathogen. Thus, we set out to functionally explore and characterize eight putative membrane proteins including MirC to further elucidate their role in iron metabolism in *A. fumigatus*.

## Methods

### Fungal strains

Strains used in this study are shown in Table S1.

### General molecular techniques

PCR reactions for generation of DNA manipulation constructs were performed using the AccuTaq LA kit (Sigma-Aldrich). For general cloning procedures the bacterial strain *Escherichia coli* DH5α was used which was cultivated in LB (1% (w/v) Bacto-tryptone, 0.5% (w/v) yeast extract, [1% (w/v) NaCl, pH 7.5] medium. Genomic DNA was extracted using the Zymogen Fungal DNA Extraction Kit (Zymo Research Corporation, USA).

### Deletion and complementation of *A. fumigatus* iron-responsive genes

The bipartite marker technique (Nielsen et al., 2006) was used to generate gene deletion strains in either *A. fumigatus* ATCC46645 or Afs77. For each deletion, either *A. fumigatus* strain was co-transformed with two DNA constructs, each containing an incomplete fragment of a pyrithiamine resistance gene (*ptrA*) (Kubodera et al., 2002) fused to 1 kb of up and downstream sequences which flanked the regions to be deleted. Fragments shared a 557 bp overlap within *ptrA*, serving as a potential recombination site during transformation. Two rounds of PCR generated each fragment. First, each flanking region was amplified from ATCC46645 genomic DNA using the primers ending with P1 and P2 for flanking region A, and P3 and P4 for flanking region B (Table S2). Subsequent to gel-purification, the fragments were digested with their corresponding restriction enzymes. The *ptrA* selection marker was released from plasmid pSK275 (a kind gift from Professor Sven Krappmann, Erlangen, Germany) by digestion with corresponding restriction enzymes, and ligated with the two flanking regions A and B described above. Two overlapping fragments were amplified from the ligation product using primers P5 and PTRoptrA2 for fragment C and primers P6 and PTRoptrA1 for fragment D. Subsequently ATCC46645 or Afs77 was transformed simultaneously with the overlapping fragments C and D. To complement *mirC*, a PCR fragment containing the *mirC* locus, including the promoter, was amplified using primers *mirC*-comp-F-*Xho*I and *mirC*-comp-R-*Sac*I with ATCC46645 gDNA as a template. This PCR product was inserted into the pCR® 2.1-TOPO® TA vector. This vector containing the *mirC* fragment was digested with *Xho*I and *Sac*I and inserted into the *Xho*I and *Sac*I sites in the pAN7-1 plasmid, which contains the *hph* selection marker. The final vector (*mirC*-hph) was linearized with *Xho*I and transformed into *A. fumigatus* Δ*mirC*. Gene disruption at the correct locus was confirmed by Southern hybridization (wildtype: 1.79 kb, Δ*mirC*: 5.49 kb). gDNA was digested with *Mfe*I and a 3′ Digoxigenin (DIG) probe was generated using the primers *mirC*-P3 and *mirC*-P6. A single copy ectopic integration in the complemented mutant strain (Δ*mirC*^*C*1^) as well as a complemented strain containing multiple integrations (Δ*mirC*^*C*2^) was verified by Southern hybridization. Disruption of genes AFUA_7G04730 (*sit2*), AFUA_6G09980 (*ncr1*), AFUA_1G01690 (*impA*), AFUA_5G10510 (*impB*), AFUA_3G03670 (*impC*), AFUA_5G07970 (*impD*), and AFUA_6G06620 (*impE*) were also confirmed by Southern hybridization.

### *A. fumigatus* ATCC46645 *mirC::GFP* fusion and *mirC^*GFP*/*RFP*^* strain

A fragment containing *mirC* plus 1,454 bp upstream, to ensure the native promoter was included, was generated by PCR using primers *mirC*-GFP-F-*Kpn*I and *mirC*-GFP-R-*Xma*I with ATCC46645 gDNA as a template. The PCR product was digested with *Kpn*I and *Xma*I to insert into the *Kpn*I and *Xma*I sites in the eGFP vector pUCGH which contains the hygromycin gene (*hph*) as the selection marker (Langfelder et al., 2001). The *mirC::GFP* pUCGH construct was transformed into competent *E. coli* OneShot Mach1-T1 (Invitrogen) and plated on LB agar containing ampicillin (50 μg/ml). Overnight growth at 37°C resulted in colonies and plasmid DNA was prepared using Qiaprep Spin Miniprep kit (Qiagen). The final construct was linearised using *Kpn*I. *A. fumigatus* Δ*mirC* was transformed with this linearised *mirC::GFP* pUCGH construct. Single copy ectopic integration of the *mirC::GFP* (*mirC*^*GFP*^) was verified by Southern hybridization. The *mirC*^*GFP*^ strain was then transformed with the pME3857 plasmid (a kind gift from Dr. Özgür Bayram) which is a mRFP::Histone2A vector with a phleoR marker (Bayram et al., 2012), thereby generating *mirC*^*GFP*/*RFP*^.

### *A. fumigatus* growth in iron-deplete conditions

Glassware used for culturing in iron-deplete conditions was treated with 1 mM EDTA followed by HCl to remove trace iron. Minimal media (MM), contained 1% (w/v) glucose, 7 mM KCl, 2 mM MgSO_4_.7H_2_O, 11 mM KH_2_PO_4_, and trace elements as previously described (Pontecorvo et al., 1953). Following autoclaving, filter sterilized L-glutamine was added to 20 mM final concentration. For iron-replete cultures, FeSO_4_ (10 μM) was included. *A. fumigatus* ATCC46645 and Afs77 cultures at 10^6^ conidia/ml were grown at 200 rpm, 37°C for up to 72 h. Plate assays were performed on MM agar with 5 × 10^3^ conidia inoculum per spot. FeSO_4_ was included at 10 μM and 10 mM to represent iron-replete and –excess, respectively. Iron starvation was enhanced with the addition of iron chelator bathophenanthroline disulfonate (BPS; 400 μM), unless otherwise stated. Hydrogen peroxide was included at 1 and 2 mM to represent oxidative stress-inducing conditions. Plates were incubated at 37°C and colony diameters measured at 48 h. Statistical analysis was carried out using Student's *t*-test where mutant strains were compared to the wild-type (WT) strain.

### Siderophore detection

Following preliminary analysis for siderophore production using the SideroTec assay (http://www.emergenbio.com, Ireland), culture supernatants from iron-deplete and iron-replete cultures of *A. fumigatus* were analyzed by RP-HPLC for the detection of extracellular siderophores at 254 nm. Supernatants were brought to 1.5 mM FeSO_4_ and ferrated siderophores were detected by absorbance at 440 nm (λ_max_ of ferrated siderophores). Peaks associated with FSC^+Fe^ and TAFC^+Fe^ were collected and identity confirmed via LC-MS/MS. The analysis of intracellular siderophores was adapted from Szigeti et al. (2014) and Winkelströter et al. (2015). Briefly, mycelia from 72 h cultures were harvested and lyophilized in triplicate. Mycelia (50 mg) from each of the strains was added to 1,000 μl of deionized H_2_O and homogenized by bead beating for 10 min using tungsten beads. Lysates were centrifuged for 10,000 g for 10 min and supernatants (200 μl) removed and ferrated to a final concentration 1.5 mM FeSO_4_.

### FSC^+Fe^ and TAFC^+Fe^ uptake experiments

WT, Δ*mirC*, and Δ*mirC*^*C*2^ strains were grown in iron-deplete MM for 21 h at 37°C (*n* = 3 each, 50 ml). Mycelia were removed using miracloth and washed with 100 ml of iron-deplete media followed by the removal of excess moisture using sterile tissue. The mycelia were wet-weighed under sterile conditions and an equal amount of mycelia was placed in each flask containing fresh iron-deplete media (50 ml). FSC^+Fe^ (Final concentration 3.8 μM) or TAFC^+Fe^ (Final concentration 25 μM) were spiked into each of the cultures and 1 ml aliquots of the supernatants were taken every hour for 3 h. Supernatants were brought to 1.5 mM FeSO_4_ and FSC^+Fe^ or TAFC^+Fe^ uptake was analyzed by RP-HPLC.

### RP-HPLC analysis

RP-HPLC analysis was carried out using an Agilent Series 1200 HPLC System with a diode array detector (DAD) and separation across a water:acetonitrile gradient with 0.1% (v/v) TFA. For analysis and quantification of siderophores, a gradient condition of 5–55% acetonitrile over 22 min at 2 ml/min was used on a C18 column (Agilent Zorbax Eclipse XDB-C18 Semi- Preparative; 5 μm particle size; 9.4 × 250 mm) with DAD detection at 254 and 440 nm. Peaks associated with extracellular siderophores were collected and identity confirmed via LC-MS/MS.

### Siderophore and protein mass spectrometry

LC-MS/MS analysis of RP-HPLC purified siderophores was carried out using an Agilent 6340 Ion Trap LC-MS System (Agilent Technologies, Santa Clara, CA). RP-HPLC purified siderophores were applied to a Zorbax SB-C18 HPLC-Chip with a 40 nl enrichment column and a 75 μm × 43 mm (5 μm particle and 300 Å pore size) analytical column (Collins et al., 2013; Owens et al., 2014; Moloney et al., 2016). For label-free quantitative (LFQ) comparative proteomics, *A. fumigatus* WT, Δ*mirC, or* Δ*mirC*^2^ were cultured for 72 h in either iron-deplete or iron-replete conditions (*n* = 4 for iron-deplete samples, *n* = 3 for iron-replete samples). Mycelial lysates were prepared in lysis buffer (100 mM Tris-HCL, 50 mM NaCl, 20 mM EDTA, 10% (v/v) glycerol, 1 mM PMSF, 1 μg/μl pepstatin A, pH 7.5) with grinding, sonication and clarified using centrifugation. The protein lysates were then precipitated using TCA/acetone and resuspended in 6 M urea, 2M thiourea, 0.1 M Tris-HCL (pH 8). After treatment with DTT and IAA, for reduction and alkylation respectively, sequencing grade trypsin combined with ProteaseMax surfactant was added (Collins et al., 2013). Digested samples were desalted prior to analysis using Zip Tips with C18 resin (Millipore). The desalted samples were analyzed via a Thermo Fisher Q-Exactive mass spectrometer coupled to a Dionex RSLC nano. LC gradients ran from 14 to 35% B (A: 0.1% (v/v) formic acid, B: 80% (v/v) acetonitrile, 0.1% (v/v) formic acid) over 2 h, and data was collected using a Top 15 method for MS/MS scans (O'Keeffe et al., 2014).

### Data analysis for proteomic profiling

LFQ comparative proteomic data analysis was carried out using MaxQuant (version 1.3.0.5), as previously described (O'Keeffe et al., 2014), with LFQ algorithm engaged for protein ratio determination. Database searching was performed using Andromeda, against a protein database consisting of *A. fumigatus* Af293 and A1163 strains. Protein and peptide FDRs were set to 1%, with a reverse database search used. Perseus (version 1.4.1.3) was used for the quantitative analysis with a minimum of 2 peptides per protein accepted for identification. Results were further filtered to include only proteins identified from a minimum of 3 replicates from either sample set. Qualitative results were generated based on unique protein detection in either condition. This was dependant on the detection of a protein in 3 biological samples from one condition, and absence of detection in all replicates of the second condition. Functional enrichment analysis of proteins was performed using the application FungiFun2 (https://elbe.hki-jena.de/fungifun/fungifun.php) with Gene Ontology (GO) and Functional Categories (FunCat) derived ontologies (Priebe et al., 2015).

### Culture conditions for microscopy

Conidia of WT, *mirC*^*GFP*^, and Δ*mirC*^*GFPRFP*^ (4 × 10^5^ per well in a 6 well plate) strains were inoculated into 4 ml of either iron-replete MM or iron-deplete MM containing BPS (400 μM), with each glass well containing a coverslip. Plates were incubated statically at 37°C for 48 h. Mycelia were visualized by both differential interference microscopy and fluorescence microscopy using an Olympus Fluo View 1,000 Laser Scanning microscope, as per Dolan et al. (2017).

### RNA extraction and mRNA isolation

RNA was isolated from mycelia, ground to a fine powder in liquid N_2_ using the RNeasy™ Plant Mini kit (Qiagen), according to the manufacturer's instructions.

### Reverse transcription quantitative PCR (RT-qPCR)

RNA was DNase treated using a DNase kit supplied by Sigma-Aldrich. cDNA synthesis was performed using qScript™ cDNA SuperMix (Quanta Biosciences) following the kit instructions. The constitutively expressed gene *A. fumigatus* calmodulin (calm) (Burns et al., 2005) was used as a reference gene. RT-qPCR was performed on the Light-Cycler® 480 Real-Time PCR System using the Light-Cycler® Sybr Green 1 Master Mix (Roche) as described previously (O'Hanlon et al., 2011). Three biological replicates for each treatment and three technical replicates for each biological replicate were used for the qRT-PCR analysis.

### *Galleria mellonella* infection experiments

*G. mellonella* larvae (*n* = 30) were inoculated into the hind pro-leg with a 20 μl inoculum volume of 5 × 10^6^ conidia. Mortality, defined by lack of movement in response to stimulation and discolouration (melanization) rates were recorded at 24 h intervals up to 96 h after injection. Kaplan-Meier survival curves were analyzed using the Log-Rank (Mantel-Cox) test for significance.

## Results

### Selection of genes to further study the ability of *A. fumigatus* to survive in iron-deplete conditions

Proteins with differential abundance under iron starvation were identified in microsomal preparations of *A. fumigatus* (Moloney et al., 2016). Target proteins with elevated abundance under iron-deplete conditions, in particular putative transporters, were selected for further characterisation using gene knockout strategies (Table 1). Proteins without previous assignation were designated Imp (Iron-modulated protein). Successful gene deletion of transporters was confirmed using Southern hybridization (Figures S1A,B, S2). *mirC* is predicted to encode a siderophore transporter of 611 amino acid residues with 12 membrane spanning domains. Residues 65–253 and 411–506 align to that of a functional family termed “siderophore iron transporter 1” which includes TAFC^+Fe^ transporter, MirB using CATH (http://www.cathdb.info) (Haas et al., 2003; Raymond-Bouchard et al., 2012; Sillitoe et al., 2015). Absence of *mirC* expression in *A. fumigatus* Δ*mirC*, and restoration of expression in Δ*mirC*^*C*2^ and *mirC*^*GFP*^ and was confirmed by RT-PCR (Figure S2H).

**Table 1:** **Targets for gene knockouts in ***A. fumigatus*****.

**Accession**	**Protein name**	**Description**	***p*-value**	**Log_2_ (Fold Increase)**	**Strain**
AFUA_1G01690	ImpA	Heme binding activity	0.000927	17.1	Afs77 Ku
AFUA_5G10510	ImpB	Role in transmembrane transport	4.6E-06	9.4	Afs77 Ku
AFUA_3G03670	ImpC	ABC multidrug transporter	0.000257	2.9	ATCC46645
AFUA_2G05730	MirC	Putative siderophore transporter	3.44E-06	1.9	ATCC46645
AFUA_6G09980	Ncr1	Putative sphingolipid transporter	0.00631	1.0	Afs77 Ku
AFUA_7G04730	Sit2	Putative siderophore transporter	NA	Unique	Afs77 Ku
AFUA_5G07970	ImpD	Bilirubin transmembrane transporter activity	NA	Unique	Afs77 Ku
AFUA_6G06620	ImpE	Role in ER to Golgi vesicle-mediated transport	NA	Unique	Afs77 Ku

*Proteins with significantly increased abundance in the A. fumigatus microsome in response to iron starvation (p < 0.05), as identified by LFQ comparative quantitative mass spectrometry with Progenesis LC-MS analysis (Moloney et al., 2016)*.

### Growth of *ΔmirC* is reduced during iron limitation

**Figure 1:**
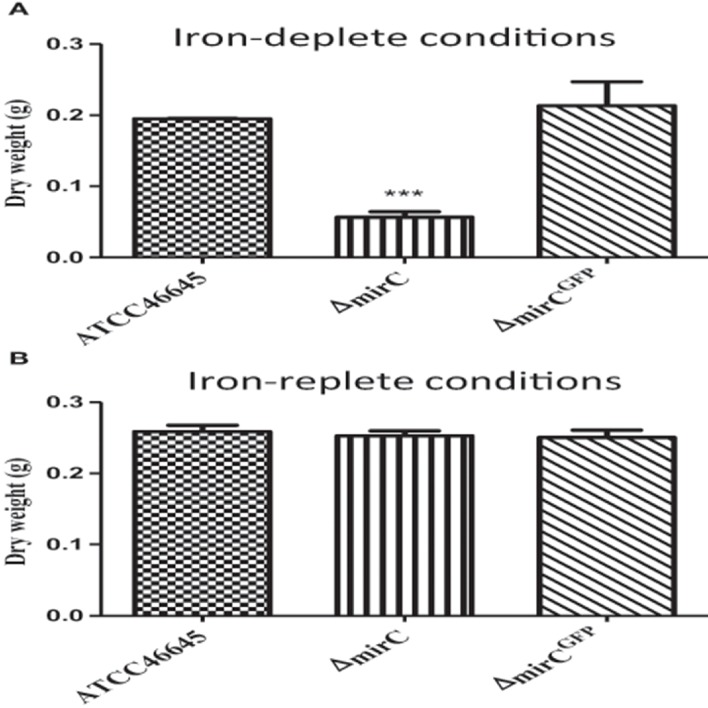
***A. fumigatus*** **Δ***mirC*** is sensitive to iron-deplete conditions**. Dry weight of *A. fumigatus* WT, Δ*mirC* and Δ*mirC*^*GFP*^ grown in **(A)** iron-deplete MM or **(B)** iron-replete MM for 72 h at 37°C. The dry weight of Δ*mirC* was significantly decreased (*p* < 0.001) compared to the WT while there was no statistical difference between the WT and Δ*mirC*^*GFP*^. There was no difference in dry weight between the strains in iron-replete MM. Statistical analyses were carried out using *t*-tests. (^***^p < 0.001).

**Figure 2:**
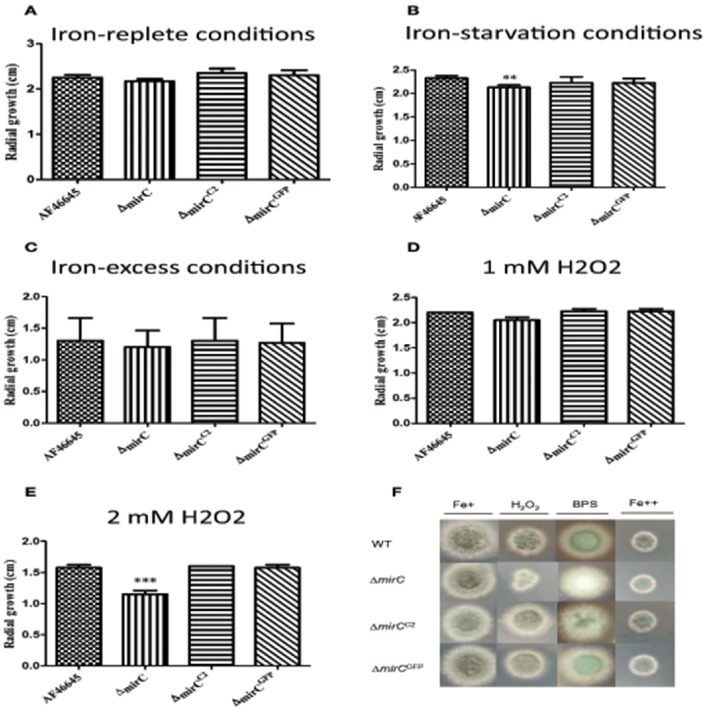
**Phenotypic analysis of ***A. fumigatus*** ATCC46645, Δ***mirC***, Δ***mirC***^***C***2^, and Δ***mirC***^***GFP***^ strains**. The radial growth was measured in *A. fumigatus* WT and deletion strains grown on MM plates under **(A)** iron sufficiency, **(B)** iron starvation (400 μM BPS), **(C)** iron excess (10 mM FeSO_4_), **(D)** 1 mM hydrogen peroxide, and **(E)** 2 mM hydrogen peroxide after 48 h at 37°C. Radial growth of Δ*mirC* was significantly decreased in response to iron starvation (*p* < 0.01) and oxidative stress at 2 mM hydrogen peroxide (*p* < 0.001). Δ*mirC* also displayed reduced conidiation compared to the WT and complemented strains when exposed to BPS (400 μM). **(F)** *A. fumigatus* WT, Δ*mirC*, Δ*mirC*^*C*2^, and Δ*mirC*^*GFP*^ grown on MM plates containing iron-replete (Fe+), H_2_O_2_ (2mM), BPS (400 μM), and iron-excess (Fe++) for 48 h at 37°C. Δ*mirC* was significantly more sensitive to H_2_O_2_ compared to the WT and complemented strains. Δ*mirC* also has decreased conidiation compared to the WT and complemented strain when exposed to BPS (400 μM). ^**^p < 0.01; ^***^p < 0.001.

The dry weight of Δ*mirC* was significantly (*p* < 0.001) decreased at 72 h compared to WT, while there was no significant difference between *mirC*^*GFP*^ and WT under iron-deplete conditions (Figure 1). During iron-replete growth, no difference between the dry weights of the three strains was observed (Figure 1). To confirm that Δ*mirC* growth was impeded under iron limitation, assays were carried out on MM plates containing the iron chelator BPS (400 μM). Δ*mirC* exhibited reduced conidiation and significantly decreased radial growth (*p* < 0.01) compared to the WT and complemented strains (Δ*mirC*^*C*2^ and Δ*mirC*^*GFP*^) under iron starvation while no difference was observed under iron-replete or iron-excess conditions (Figure 2). There was no significant difference in the dry weights under iron-deplete conditions or radial growth under iron-replete, iron starvation or iron-excess conditions of Δ*sit2* (AFUA_7G04730), Δ*ncr1* (AFUA_6G09980), Δ*impA* (AFUA_1G01690), Δ*impB* (AFUA_5G10510), Δ*impC* (AFUA_3G03670), Δ*impD* (AFUA_5G07970), and Δ*impE* (AFUA_6G06620) compared with the relevant WT strains (Figures S3, S4).

### *A. fumigatus ΔmirC* is more sensitive to hydrogen peroxide

Growth of Δ*mirC* in 2 mM hydrogen peroxide was significantly reduced compared to the WT (*p* < 0.001), while there was no difference between the WT and the complemented strains (Δ*mirC*^*C*2^ and Δ*mirC*^*GFP*^) (Figure 2). There was no significant difference in the radial growth of Δ*sit2*, Δ*ncr1*, Δ*impA*, Δ*impB*, Δ*impC*, Δ*impD*, and Δ*impE* compared with the relevant WT strains under different concentrations of hydrogen peroxide (Figures S3D,E, S4D,E).

### Differential extracellular siderophore levels in Δ*impC* but not Δ*mirC* under iron limitation

**Figure 3:**
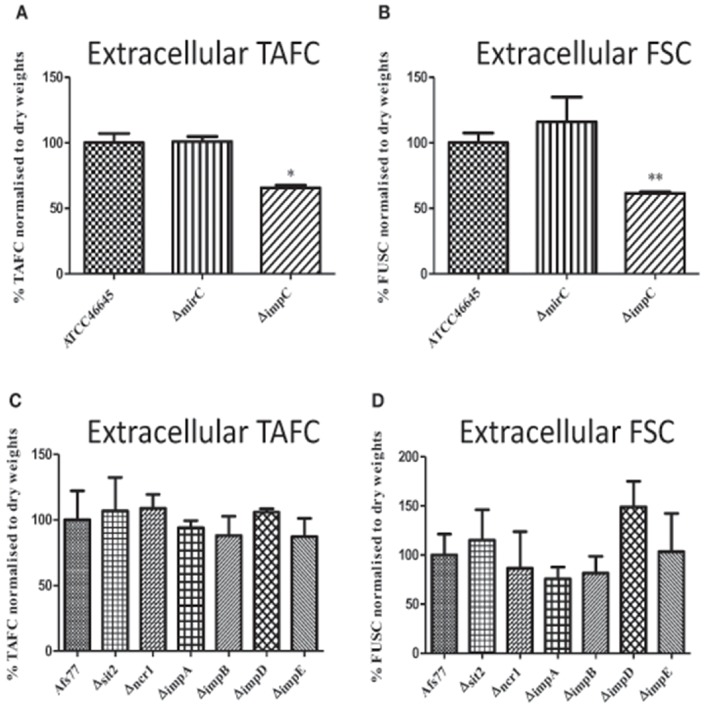
**Extracellular siderophore production in ***A. fumigatus*** deletion strains**. RP-HPLC analysis was used to measure extracellular siderophore levels in *A. fumigatus* WT and transporter knock-out strains grown in iron-deplete conditions for 72 h at 37°C (*n* = 3). Siderophore levels were normalized to biomass. Supernatant was ferrated at 1.5 mM FeSO_4_ before the analysis. Levels of **(A)** TAFC^+Fe^ and **(B)** FSC^+Fe^ were decreased in Δ*impC* while no significant difference was observed in siderophore levels for **(C,D)** Δ*mirC* or other knock-out strains. ^*^p < 0.05; ^**^p < 0.01.

Extracellular siderophore levels from WT, Δ*mirC*, Δ*impC*, and Δ*impD* grown under iron limitation for 72 h were analyzed by RP-HPLC. Immediately prior to analysis, supernatants were brought to 1.5 mM FeSO_4_. Siderophore levels were normalized to biomass and the identity of peaks associated with FSC^+Fe^ and TAFC^+Fe^ was confirmed via LC-MS/MS (Figure S5). There was no significant difference in the level of extracellular siderophores produced by Δ*mirC* at 72 h (Figures 3A,B). Interestingly, Δ*impC* showed a significant decrease in levels of TAFC^+Fe^ (*p* < 0.05) and FSC^+Fe^ (*p* < 0.01) (Figures 3A,B). Finally, there was no significant difference in the levels of TAFC^+Fe^ or FSC^+Fe^ secreted by Δ*ncr1*, Δ*sit2*, Δ*impA*, Δ*impB*, Δ*impD*, Δ*impE* compared to the WT strain, under iron-deplete conditions (Figures 3C,D). There was no significant difference in the uptake of TAFC^+Fe^ and FSC^+Fe^ between WT, Δ*mirC*, and Δ*mirC*^*C*2^ at the measured time-points (Figure S7). This suggests that deletion of *mirC* does not significantly alter the rate of uptake of TAFC^+Fe^ or FSC^+Fe^.

### Intracellular ferricrocin level is significantly decreased in Δ*mirC* mycelia under iron limitation

**Figure 4:**
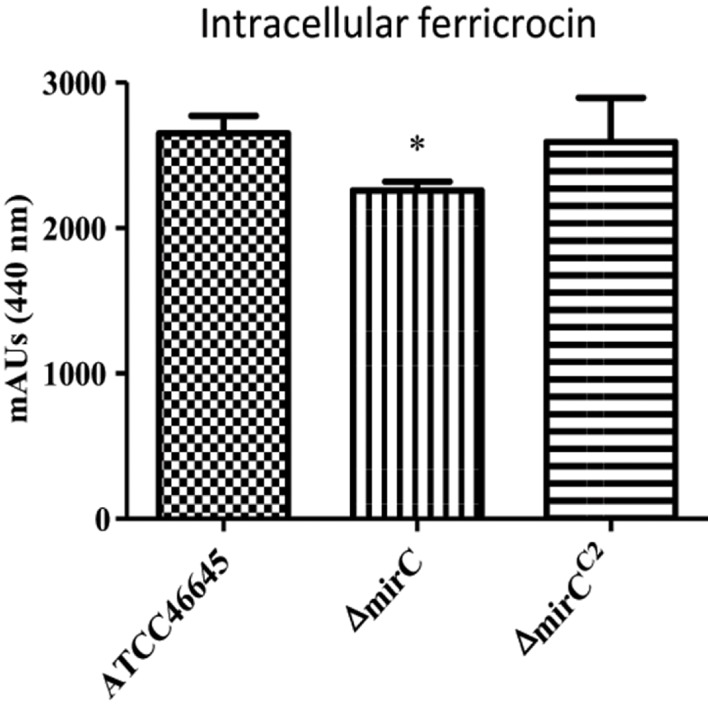
**Intracellular siderophore content reduced in Δ***mirC*** during iron-deplete growth**. FC content of dried mycelia was analyzed by RP-HPLC. Ferricrocin was significantly reduced in Δ*mirC* vs. WT and Δ*mirC*^*C*2^ mycelia when grown in MM for 72 h at 37°C (*p* < 0.05). Statistical analyses were carried out using t-tests. (^*^p < 0.05).

Mycelia from the WT, Δ*mirC*, and Δ*mirC*^*C*2^ were harvested after 72 h in iron-deplete conditions (*n* = 4). Lysed mycelial supernatants were ferrated to a final concentration of 1.5 mM FeSO_4_ before RP-HPLC analysis at 440 nm. The identity of the peak associated with FC identity was also confirmed by LC-MS/MS (Figure S5). Intracellular FC was found to be significantly reduced in the mycelia of Δ*mirC* vs. the WT and Δ*mirC*^*C*2^ under iron-deplete conditions (*p* < 0.05) (Figure 4). However, there was no significant difference in the amount of FC in Δ*sit2*, Δ*ncr1*, and Δ*impA*-*E* under iron-deplete conditions, compared to WT (Figures S3, S6).

### Increased extracellular siderophore levels in Δ*mirC* under iron sufficiency

**Figure 5:**
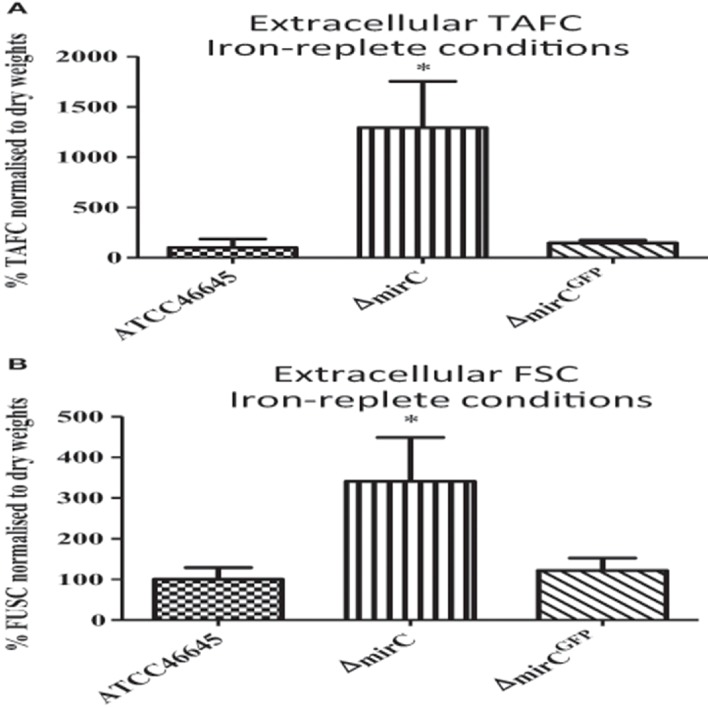
**Extracellular siderophore levels in Δ***mirC*** are increased during iron-replete growth**. RP-HPLC analysis was used to measure extracellular siderophore levels in *A. fumigatus* WT, Δ*mirC*, and Δ*mirC*^*GFP*^ grown in iron-replete conditions for 72 h at 37°C (*n* = 4). Siderophore levels were normalized to biomass. Supernatant was ferrated at 1.5 mM FeSO_4_ before the analysis. There was a significant increase in the levels of **(A)** TAFC^+Fe^ (*p* < 0.05) and **(B)** FSC^+Fe^ (*p* < 0.05) in Δ*mirC* compared to WT. Statistical analyses were carried out using t-tests (^*^p < 0.05).

Supernatants from WT, Δ*mirC*, and *mirC*^*GFP*^, grown under iron sufficiency for 72 h, were analyzed by RP-HPLC at 440 nm (*n* = 3). Prior to analysis supernatants were brought to 1.5 mM FeSO_4_. Siderophore levels were normalized to biomass. Surprisingly, there was a significant increase in the levels of TAFC^+Fe^ (*p* < 0.05) and FSC^+Fe^ (*p* < 0.05) produced by Δ*mirC*, compared to WT (Figure 5).

### Label-free quantitative proteomic analysis reveals significant alterations to the proteome of Δ*mirC* compared to the WT during both iron-deplete and -replete growth

**Figure 6:**
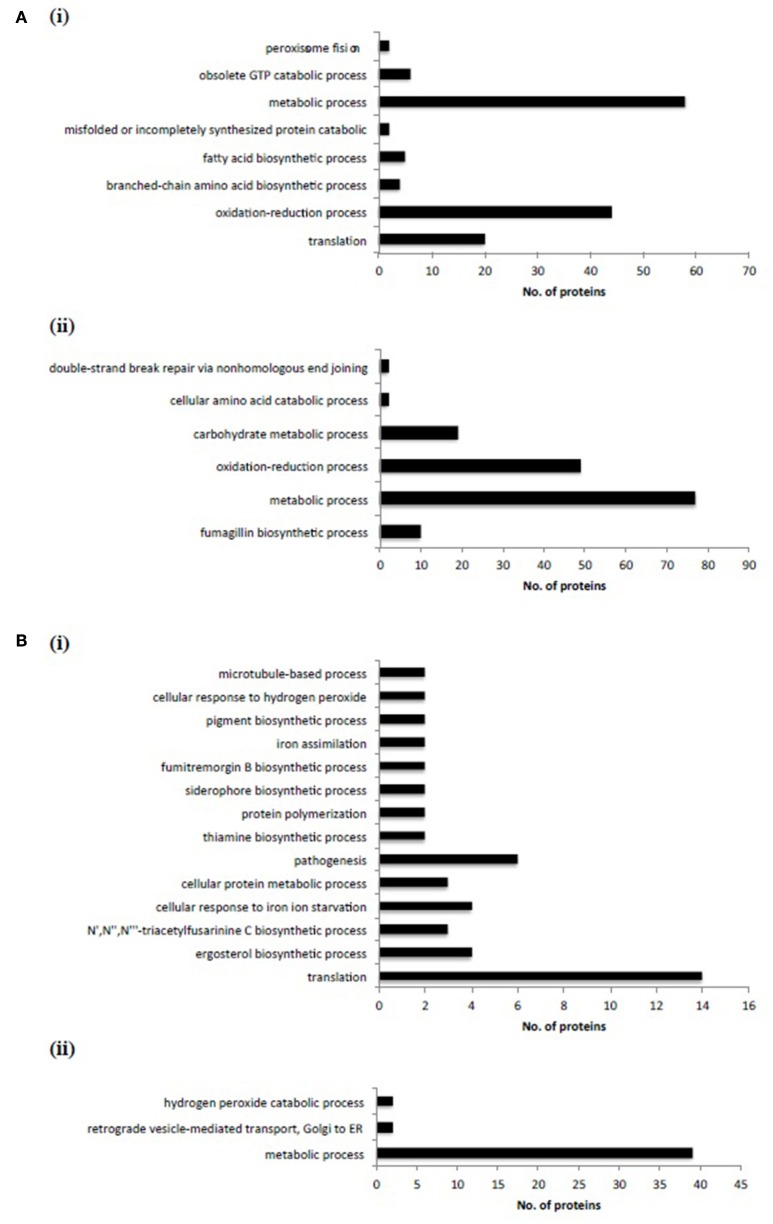
**Label-free proteomic analysis revealed significant proteomic remodeling in Δ***mirC*** compared to WT during iron-replete and –deplete growth. (A)** GO biological processes significantly enriched in proteins with (i) increased and (ii) decreased abundance during iron-replete growth in Δ*mirC* compared to WT. **(B)** GO biological processes significantly enriched in proteins with (i) increased and (ii) decreased abundance during iron-deplete growth in Δ*mirC* compared to WT. Notably, the GO biological process of “translation” was significantly represented in proteins with increased abundance during iron-replete and –deplete growth.

High sensitivity LFQ proteomic analysis revealed 511 proteins with differential abundance were identified in Δ*mirC* compared with WT during iron limitation (*n* = 4; 72 h; Figure 6); 251 showing increased abundance and 260 decreased abundance (*p* < 0.05, log_2_ fold change > 1) (Tables S4, S5). In addition, 297 proteins showed differential abundance in Δ*mirC* compared with WT during iron-replete growth (*n* = 3); 150 with increased abundance and 147 with decreased abundance (*p* < 0.05, log_2_ fold change > 1) (Tables S8, S9).

Differentially abundant proteins in Δ*mirC* compared with WT were analyzed for functional category enrichment (*p* < 0.05) using FungiFun2 with GO classification ontology (Priebe et al., 2015). GO biological process categories significantly represented by proteins with increased abundance in Δ*mirC* compared with WT during iron-deplete growth included; translation (*n* = 20), oxidation-reduction process (*n* = 44), branched-chain amino acid biosynthetic process (*n* = 4), fatty acid biosynthetic process (*n* = 5), misfolded, or incompletely synthesized protein catabolic process (*n* = 2), metabolic process (*n* = 58), obsolete GTP catabolic process (*n* = 6), and peroxisome fission (*n* = 2) (Figure 6). GO biological process categories significantly represented by proteins with decreased abundance in Δ*mirC* compared with WT during iron-deplete growth included; fumagillin biosynthetic process (*n* = 10), metabolic process (*n* = 77), oxidation-reduction process (*n* = 49), carbohydrate metabolic process (*n* = 19), cellular amino acid catabolic process (*n* = 2), and double-strand break repair via non-homologous end joining (*n* = 2) (Figure 6). Proteomic analysis also revealed increased abundance of proteins involved in siderophore biosynthesis in Δ*mirC* compared to WT during iron-deplete growth, including L-ornithine-*N*^5^-monooxygenase, SidA (AFUA_2G07680) and SidI (AFUA_1G17190) (Table 2). There was also a decreased abundance of TAFC transporter MirB (AFUA_3G03640) and putative transporter AFUA_3G03670 (ImpC) in Δ*mirC*, compared to WT (Table 3). GO biological process categories significantly represented by proteins with increased abundance in Δ*mirC* compared with WT during iron-replete growth included; translation (*n* = 14), ergosterol biosynthetic process (*n* = 4), N′,N″,N‴-triacetylfusarinine C biosynthetic process (*n* = 3), cellular response to iron starvation (*n* = 4), cellular protein metabolic process (*n* = 3), pathogenesis (*n* = 6), thiamine biosynthetic process (*n* = 2), protein polymerization (*n* = 2), siderophore biosynthetic process (*n* = 2), fumitremorgin B biosynthetic process (*n* = 2), iron assimilation (*n* = 2), pigment biosynthetic process (*n* = 2), cellular response to hydrogen peroxide (*n* = 2), and microtubule-based process (*n* = 2) (Figure 6). GO biological process categories significantly represented by proteins with decreased abundance in Δ*mirC* compared with WT during iron-replete growth included; metabolic process (*n* = 39), retrograde vesicle-mediated transport (*n* = 2), and hydrogen peroxide catabolic process (*n* = 2) (Figure 6).

**Table 2:** **Proteins associated with siderophore biosynthesis and transporter activity with increased abundance in ***A. fumigatus*** Δ***mirC*** mycelia grown for 72 h in iron-deplete MM compared to ***A. fumigatus*** WT (See also Tables S3, S6, S7, S10 and S11)**.

**Protein description**	**Log_2_ (Fold Increase)**	***p*-value**	**Peptides**	**Sequence coverage [%]**	**Protein IDs**
Ortholog(s) have iron ion transmembrane transporter activity	Unique	N/A	4	33.7	AFUA_6G12550
Ortholog(s) have guanine nucleotide transmembrane transporter activity, role in cellular iron ion homeostasis	1.54	5.53E-04	17	56.6	AFUA_1G07450
L-ornithine N5-oxygenase, first committed step in siderophore biosynthesis. **SidA**.	1.47	4.72E-06	48	83.2	AFUA_2G07680
Cytochrome c; transcript derepressed during iron starvation in hapX mutant	1.02	6.79E-03	5	14	AFUA_2G13110
Putative peroxisome biogenesis factor	1.32	1.70E-03	5	26.8	AFUA_6G07740
Putative peroxin with a predicted role in peroxisome biogenesis	1.14	4.34E-03	3	12	AFUA_2G17100
Putative long-chain-fatty-acid-CoA ligase. **SidI**.	1.09	5.69E-04	38	75.8	AFUA_1G17190

**Table 3:** **Proteins associated with siderophore biosynthesis and transporter activity with decreased abundance in ***A. fumigatus*** Δ***mirC*** mycelia grown for 72 h in iron-deplete MM compared to ***A. fumigatus*** WT (See also Tables S3, S6, S7, S10 and S11)**.

**Protein description**	**Log_2_(Fold Decrease)**	***p*-value**	**Peptides**	**Sequence coverage [%]**	**Protein IDs**
Putative siderophore iron transporter;SrbA-regulated during hypoxia. **MirB**.	−1.55	1.04E-03	9	26.1	AFUA_3G03640
ABC multidrug transporter with a predicted role in iron metabolism;part of an iron-regulated gene cluster. **ImpC**.	−1.84	5.55E-03	7	9.7	AFUA_3G03670

Several GO-enriched categories representing proteins with increased abundance during iron-replete growth in Δ*mirC* compared with WT were also observed in WT with increased abundance in response to iron starvation. These categories included, translation, cellular protein metabolic process, siderophore biosynthetic process, ergosterol biosynthetic process, and N′,N″,N‴-triacetylfusarinine C biosynthetic process (Tables S8, S12). Interestingly, specific proteins associated with siderophore biosynthesis and transport functions were among these (Table 4).

**Table 4:** **Proteins associated with siderophore biosynthesis and transporter activity with increased abundance in ***A. fumigatus*** Δ***mirC*** mycelia grown for 72 h in iron-replete MM compared to ***A. fumigatus*** WT (See also Tables S3, S6, S7, S10 and S11)**.

**Protein description**	**Log_2_ (Fold Increase)**	***p*-value**	**Peptides**	**Sequence coverage [%]**	**Protein IDs**
Putative long-chain-fatty-acid-CoA ligase. **SidI**.	Unique	N/A	38	75.8	AFUA_1G17190
Putative siderophore biosynthesis lipase/esterase. **EstB**.	Unique	N/A	9	58.9	AFUA_3G03660
L-ornithine N5-oxygenase, first committed step in siderophore biosynthesis. **SidA**.	6.42	2.55E-04	48	83.2	AFUA_2G07680
Putative siderophore iron transporter;SrbA-regulated during hypoxia. **MirB**.	3.48	8.17E-03	9	26.1	AFUA_3G03640
Putative siderophore transporter;expression upregulated under low iron conditions. **MirD**.	2.35	1.71E-03	7	13.2	AFUA_3G03440
Hydroxyornithine transacylase;involved in extracellular siderophore biosynthesis;essential for triacetylfusarinine C biosynthesis. **SidF**.	2.01	1.76E-04	38	90	AFUA_3G03400
Putative siderophore transporter;SrbA-regulated during hypoxia. **Sit1**.	1.73	4.72E-02	5	11.4	AFUA_7G06060
Putative siderophore biosynthesis lipase/esterase. **SidJ**.	1.15	4.65E-03	15	66.7	AFUA_3G03390

### *hapX* expression is up-regulated in Δ*mirC* under iron-replete but not iron-deplete conditions compared to WT

**Figure 7:**
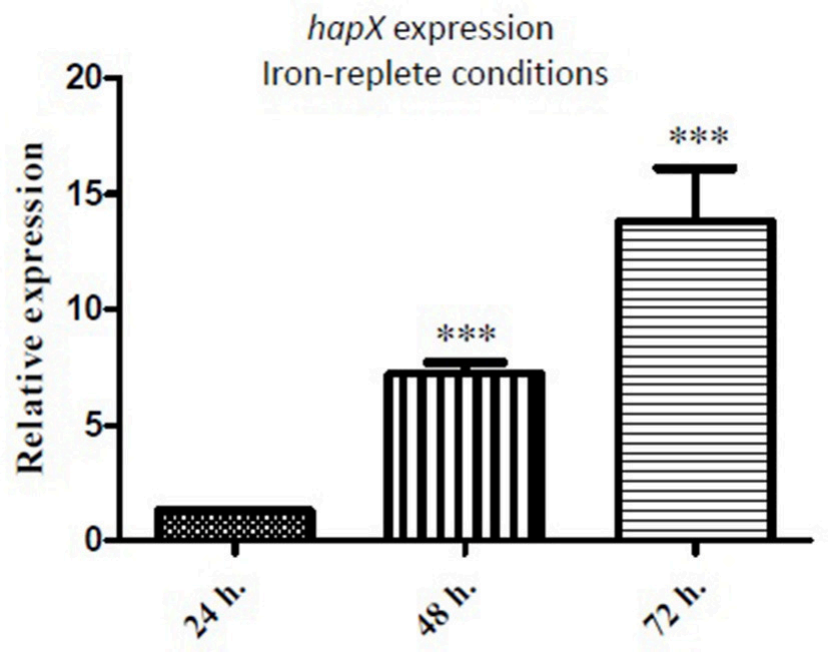
***hapX*** **is up-regulated in Δ***mirC*** during iron-replete growth**. qRT-PCR analysis of *hapX* expression in Δ*mirC* vs. WT after growth under iron-deplete or iron-replete minimal media for 72 h. *hapX* expression was significantly up-regulated (*p* < 0.001) in Δ*mirC* under iron-replete but not iron-deplete conditions. ^***^p < 0.001.

qRT-PCR analysis of *A. fumigatus hapX* expression in the Δ*mirC* and WT strains was carried out after growth in iron-deplete or iron-replete minimal media for 24–72 h (Figure 7). *hapX* expression was significantly up-regulated (*p* < 0.001) in Δ*mirC* (7.2-fold at 48 h and 13.8-fold at 72 h) under iron-replete conditions. *hapX* expression was not significantly different between the Δ*mirC* and WT strains under iron-deplete conditions (72 h; data not shown), or at 24 h under iron-replete conditions.

### *mirC* appears to exhibit an intracellular localization

**Figure 8:**
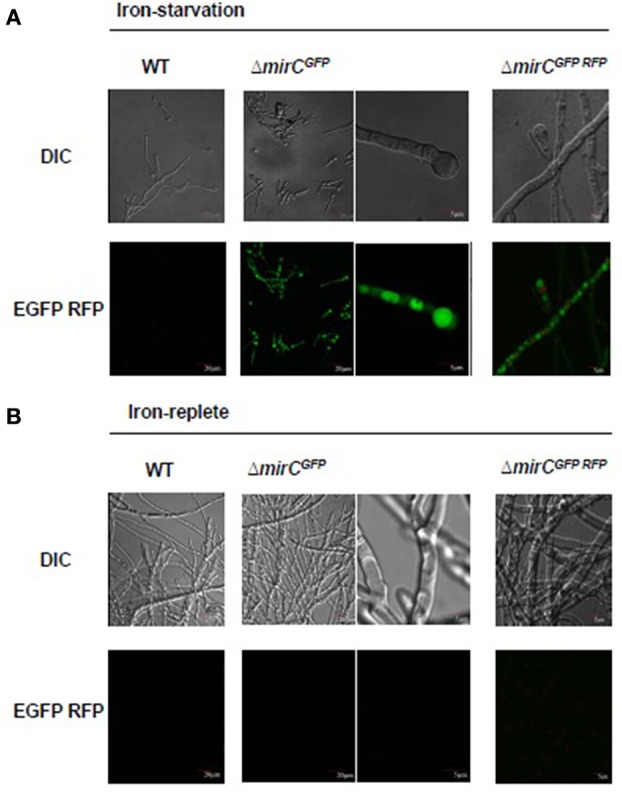
**MirC exhibits intracellular localization during iron-deplete but not –replete growth**. WT, Δ*mirC*^*GFP*^, and Δ*mirC*^*GFPRFP*^ were grown in **(A)** iron-starved (400 μM BPS) and **(B)** iron–replete conditions for 48 h at 37°C. Hyphae were observed by differential interference microscopy (DIC). Fluorescence was localized intracellularly, possibly to vacuoles, in Δ*mirC*^*GFP*^, and Δ*mirC*^*GFPRFP*^ during iron-starved but not –replete growth.

MirC-GFP fusion strain fluorescence was only evident under iron starvation, not iron sufficiency, in *mirC*^*GFP*^ (Figure 8), and was localized intracellularly, possibly to vacuole-like structures. There was no evidence of MirC-GFP presence in the plasma membrane, although it is not possible to definitively assign the intracellular localization of the fusion protein, due to apparent degradation-even though intact MirC-GFP was also detected by Western blot analysis (Figure S8). Moreover, no fluorescence was observed in the WT control under iron-replete or iron-deplete conditions. *mirC*^*GFP*^ was also transformed to create a MirC::GFP mRFP::Histone2A fusion strain to further elucidate the location of MirC-GFP expression. MirC-GFP fluorescence did not co-localize with mRFP::Histone2A fluorescence, which indicates that it is not localized to nuclei (Figure 8).

### Disruption of *mirC* decreases virulence in *G. mellonella*

**Figure 9:**
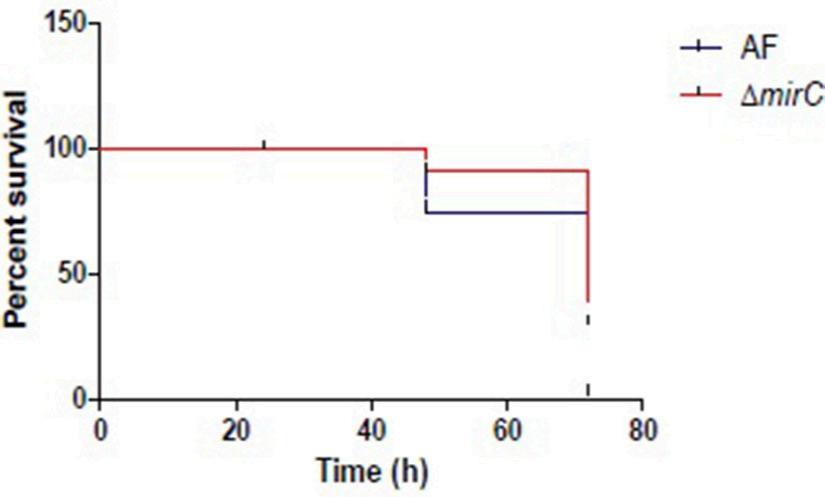
**Δ***mirC*** exhibits decreased virulence in ***G. mellonella*****. The survival curves of *G. mellonella* larvae when infected with 5 × 10^6^ conidia per insect for ATCC46645 and Δ*mirC*. The Mantel-Cox test revealed that the virulence is significantly reduced in Δ*mirC* vs. the WT strain (*p* < 0.002).

The virulence of Δ*mirC* in *G. mellonella* was significantly reduced compared to the WT strain (Figure 9). The Mantel-Cox test revealed that the survival curves of WT and Δ*mirC* were significantly different (*p* < 0.002).

## Discussion

Despite significant efforts (Haas et al., 2003; Philpott and Protchenko, 2008; Moloney et al., 2016), little is known about the transporters involved in siderophore–mediated iron acquisition. Consequently, we functionally investigated putative siderophore transporters identified in previous proteomic analysis (Moloney et al., 2016). Deletion of *mirC* in *A. fumigatus* significantly decreased growth under iron-depletion and oxidative stress conditions, reduced intracellular ferricrocin levels, and attenuated virulence in the *G. mellonella* model. Additionally, LFQ proteomic analysis of Δ*mirC* revealed an increased abundance of proteins involved in siderophore biosynthesis in both iron-replete and -deplete conditions, indicating a dysregulated iron-sensing capacity. MirC appears to be located intracellularly in *A. fumigatus*. Interestingly, there was no decrease in growth under iron-deplete or oxidative conditions in the other deletion strains presented herein.

Significantly increased abundance of MirC was identified in the microsomal proteome of *A. fumigatus* under iron starvation (Moloney et al., 2016). The GATA-type transcription factor, SreA which is an important transcriptional regulator of iron homeostasis represses *mirC* expression in the presence of iron (Schrettl et al., 2008). Accordingly, it appears that *mirC* is involved in iron homeostasis since reduced growth was observed during iron starvation, following *mirC* deletion (*p* < 0.001) (Figures 1, 2). Compared to WT, no significant difference in extracellular siderophore production or uptake was observed for Δ*mirC* during iron starvation, thereby excluding a direct role for this transporter in siderophore uptake or efflux. This result is in accordance with previous work by Haas et al. (2003) in which *Aspergillus nidulans mirC* ortholog was expressed in an *S. cerevisiae* strain deficient in high affinity iron acquisition mechanisms (Haas et al., 2003). Analysis of iron uptake from siderophores TAFC, FC, ferrichrome, and enterobactin in this strain did not indicate any role for *mirC* in siderophore uptake.

In contrast, levels of intracellular FC decreased in Δ*mirC* compared to WT during iron-deplete growth (*p* < 0.05) (Figure 4). FC predominantly functions in the transport and storage of intracellular iron following ferri-siderophore uptake (Schrettl et al., 2007; Wallner et al., 2009; Blatzer et al., 2011). Depletion of intracellular siderophores, FC and HFC, has previously been implicated in impaired oxidative stress tolerance in hyphae and conidia (Schrettl et al., 2007; Blatzer et al., 2011). Iron and redox homeostasis mechanisms are tightly intertwined processes in *A. fumigatus* (Brandon et al., 2015) which is largely a result of the redox potential of free iron and its requirement as a co-factor for many antioxidant enzymes. Following the reduced intracellular FC content, it is logical that Δ*mirC* also displayed decreased tolerance to hydrogen peroxide compared to WT during iron-replete growth (*p* < 0.001) (Figure 2). Interestingly, Δ*mirC* exhibited significantly increased extracellular siderophore production during iron-replete growth suggesting a dysregulation of iron homeostasis (Figure 5). Thus, for a global and in-depth analysis of the impact of *mirC* deletion in *A. fumigatus*, large-scale LFQ proteomic analysis was employed to compare Δ*mirC* to WT during both iron-deplete and -replete growth.

Differential abundance of a total of 297 proteins (*p* < 0.05) was observed during iron-replete growth in Δ*mirC* compared to WT (Tables S8, S9). Moreover, with enrichment of GO biological process categories including translation (*n* = 14), ergosterol biosynthetic process (*n* = 4), N′,N″,N‴-triacetylfusarinine C biosynthetic process (*n* = 3), and siderophore biosynthetic process (*n* = 2), the iron-replete induced proteome of Δ*mirC* actually reflected that of WT during iron-deplete growth (Figure 6; Table 4; Table S8). In fact, 18 of the proteins observed in these categories also showed increased abundance in WT during iron-deplete growth compared to iron-replete. Proteins involved in siderophore biosynthesis with increased abundance in both protein datasets included L-ornithine *N*^5^-oxygenase SidA (AFUA_2G07680). Additionally, peroxisomally located siderophore biosynthetic enzymes required for FSC and TAFC biosynthesis were also shared; mevalonyl-CoA ligase SidI (AFUA_1G17190), transacylase SidF (AFUA_3G03400), and mevalonyl-CoA hydratase SidH (AFUA_3G03410). Strikingly, 27 proteins associated with the ribosome showed differential abundance in Δ*mirC* compared with WT during iron-replete growth (Tables S8, S9). Of the 19 ribosome-associated proteins with increased abundance, 14 were also increased in WT during iron-deplete growth compared to iron-replete. Translation and ribosome biogenesis are iron-consuming processes and HapX-mediated repression of ribosome biogenesis has been observed in *A. fumigatus* during iron starvation (Schrettl et al., 2010). So, the increased abundance of ribosomal proteins during iron starvation in WT may represent a ribosomal reprogramming necessary for maintaining viability during reduced iron availability. HapX-mediated regulation of enzymes related to cellular detoxification processes has also been previously observed (Schrettl et al., 2010). Enzymatic antioxidant mechanisms including peroxidases and catalases are dependent on iron as a heme constituent. Accordingly, Δ*sidC* conidia with reduced HFC and iron content also show reduced catalase (CatA; AFUA_6G03890) activity implicated in hydrogen peroxide sensitivity. In contrast, Δ*sidC* hyphae showed no reduced catalase activity in the form of catalase 1 (Cat1; AFUA_3G02270) and catalase 2 (Cat2; AFUA_8G01670) activity (Schrettl et al., 2007). Interestingly, Cat1 and Cat2 showed decreased abundance in Δ*mirC* during iron-replete growth compared to WT and thus may at least partially underlie the impaired oxidative stress tolerance observed (Figure 2). Though there was no difference in Δ*sidC* hyphae compared with WT, Schrettl et al. (2007) reported a reduction in Cat1 and Cat2 activity between iron-deplete and –replete growth (Schrettl et al., 2007). Given that Δ*mirC* displays an iron-starved phenotype during iron-replete growth, it is likely that the decreased abundance of these proteins is a result of an iron starvation response. This is supported by HapX-mediated repression of *cat1* transcription during iron starvation (Schrettl et al., 2010). HapX is an iron-repressed bZip transcription factor that, in addition to SreA, governs iron homeostasis by inducing iron-acquiring pathways and repressing iron-requiring pathways. RT-qPCR revealed that *hapX* expression was up-regulated in Δ*mirC* between 7 and 13-fold (*p* < 0.001, at 48 and 72 h growth) during iron sufficiency (Figure 7). Thus, there is strong evidence that during iron-replete growth Δ*mirC* is sensing an iron-limiting environment.

During iron-deplete growth, differential abundance of a total of 511 proteins (*p* < 0.05) indicated significant phenotypic remodeling in Δ*mirC* compared with WT (Tables S4, S5; Figure 6; Tables 2, 3). Similar to the proteome during iron-replete growth, the GO biological process of translation was also significantly enriched (*n* = 20). A total of 35 proteins associated with the ribosome showed differential abundance in Δ*mirC* compared with WT during iron-replete growth. Of these, 29 proteins showed increased abundance, 13 of which were also increased in WT during iron-deplete growth compared to iron-replete. Interestingly, 3 translation elongation factor proteins were uniquely detected in Δ*mirC* EF-1 (AFUA_1G06390), EF-2 (AFUA_2G13530), and eEF-3 (AFUA_7G05660) compared with WT. Importantly several siderophore biosynthetic enzymes exhibited increased abundance in *mirC* compared with WT including SidA and SidI (Table 4). Together these data indicate a dysregulation of the response of *A. fumigatus* to iron limitation, following deletion of *mirC*. The consequence of this differential response to iron starvation is evident in the reduced virulence displayed by Δ*mirC* compared to WT in *G. mellonella* (*p* < 0.002) (Figure 9), where intact mechanisms for siderophore-mediated iron acquisition augment virulence (Slater et al., 2011). Significant insight into the iron-starved mycelial proteome of WT *A. fumigatus* is also apparent. Comparing iron-deplete to –replete growth in WT; 806 proteins showed increased abundance while 518 decreased (*p* < 0.05) (Tables S12, S13). Similarly, comparing iron-deplete to –replete growth in Δ*mirC*; 952 proteins showed increased abundance while 622 decreased (*p* < 0.05) (Tables S14, S15), indicating the magnitude of proteome remodeling induced under iron starvation in *A. fumigatus*.

Siderophore biosynthesis appears to be at least partially compartmentalized, with SidI-, SidH-, and SidF-catalyzed biosynthetic steps occurring in peroxisomes (Gründlinger et al., 2013b), while *N*^5^ transacylase, SidL is localized to the cytoplasm suggesting the following biosynthetic step occurs there (Blatzer et al., 2011). Cellular localization of the later biosynthetic steps awaits elucidation, including that of NRPSs, SidD, and SidC, although identification of both NRPSs from the microsomal fraction during iron starvation suggests that these proteins may either be localized at membranes or enclosed in membrane-bound compartments (Moloney et al., 2016). Such localization has been reported in *Pseudomonas aeruginosa* where the formation of “siderosomes” at membranes is postulated to increase biosynthetic efficiency (Imperi and Visca, 2013; Gasser et al., 2015). Recent work has also revealed the localization of siderophore procurement and processing mechanisms. Immunofluorescence studies with TAFC^+Fe^ transporter, MirB indicated localization to vesicles at hyphal tips and the cytoplasm during iron starvation (Raymond-Bouchard et al., 2012; Moore, 2013). Finally, fluorescently derivatised FSC^+Fe^ localized to vacuoles following re-uptake (Moloney et al., 2016). Herein, MirC-GFP showed intracellular fluorescence during iron-deplete but not –replete growth (Figure 8), moreover, *mirC*^*GFP*/*RFP*^ revealed that MirC-GFP fluorescence did not co-localise to the nucleus (Figure 8). Though immunofluorescence indicated localization of MirB in vesicles at hyphal tips (Moore, 2013; Raymond-Bouchard et al., 2012), to the best of our knowledge MirC represents the first SIT to be localized to an intracellular location. While GFP localization studies did not exhibit fluorescence during iron-replete growth, it is clear from the observable phenotype that MirC also plays a role during iron sufficiency. Notably, MirC was not uniquely abundant in the iron-deplete microsomal proteome, but showed a 3.81-fold increase in abundance (*p* < 0.05) (Moloney et al., 2016). This indicates that MirC is present during iron-replete growth which suggests that it may function during iron sufficiency. Thus, labeled MirC levels may be too low such to facilitate detection in iron-replete Δ*mirC*^*GFP*^.

Interestingly, FC/HFC deficient strains generated by deletion of either *sidL* or *sidC* display a remarkably similar phenotype to Δ*mirC*. Deletion of *sidL* or *sidC* resulted in reduced growth consequent to iron starvation and oxidative stress, as well as up-regulation of *hapX* in iron-replete conidia (Schrettl et al., 2007; Blatzer et al., 2011). Furthermore, deficiency in intracellular siderophore content in Δ*sidL* and Δ*sidC* has been associated with reduced conidiation (Blatzer et al., 2011; Schrettl et al., 2007), which is also observed for Δ*mirC* during iron-deplete growth (Figure 2). Thus, many phenotypes observed in *A. fumigatus* following *mirC* deletion may be accounted for by a deficiency in FC/HFC content. Importantly, depletion of FC content accords with the iron-replete phenotype of Δ*mirC*. FC is utilized by *A. fumigatus* during iron-deplete conditions, and at lower levels during iron–replete growth. Δ*sidL* conidia harvested during the latter condition exhibited an iron-starved phenotype, whereby up-regulated *hapX* expression was evident (Blatzer et al., 2011). Thus, despite a low abundance, we propose that MirC functions in FC biosynthesis during iron-replete, as well as iron-deplete growth. The lack of complete abrogation of FC production in Δ*mirC* likely reflects redundancy in the siderophore-related proteome.

No specific phenotype was observed for *A. fumigatus* Δ*sit2*, however it has recently been shown that Sit2 is involved in ferrioxamine B uptake (Park et al., 2016). ImpC (AFUA_3G03670) is a putative ABC transporter and mRNA expression of ImpC is induced in response to iron-deplete conditions (Kragl et al., 2007). Significantly increased abundance of ImpC was found in the microsomal fractions of iron-depleted mycelia in *A. fumigatus* (Moloney et al., 2016). Furthermore, ImpC has been found to be regulated by the iron-sensitive transcription factor SreA and was suggested to be involved in siderophore secretion (Schrettl et al., 2008). There was a significant reduction in extracellular TAFC and FSC at 72 h growth of Δ*impC* under iron-deplete conditions, and suggests a role for this ABC transporter in the export of desferri-siderophores from the cell. However, growth of Δ*impC* was unaffected under iron-deplete or oxidative stress conditions. This indicates that there may be a functional redundancy in the export of siderophores in *A. fumigatus*.

In conclusion, we have identified MirC as a putative vacuolar membrane protein, elucidated its role in ferricrocin biosynthesis and oxidative stress, and reveal dysregulation of siderophore biosynthetic systems in the absence of MirC under iron-replete conditions.

## Ethics statement

Study exempt as animals used were insect larvae (*Galleria mellonella*).

## Author contributions

EM, NM, SKD, and LR carried out experimental work. SD, EM, NM, and RO analyzed data. RO contributed methodology and proteomics expertise. EM, NM, and SD wrote the manuscript. All authors reviewed and approved the manuscript.

### Conflict of interest statement

The authors declare that the research was conducted in the absence of any commercial or financial relationships that could be construed as a potential conflict of interest.
