# Supplementary material for: Functional Investigation of Iron-Responsive Microsomal Proteins, including MirC, in Aspergillus fumigatus
Source: Front Microbiol. 2017 Mar 17;8:418. doi: 10.3389/fmicb.2017.00418 (PMC5355445; doi:10.3389/fmicb.2017.00418)
Supplement: Supplementary file 2 [file DataSheet2.docx]

**Running head:** MirC role in fungal iron homeostasis.

**Functional Investigation of Iron Responsive Microsomal Proteins, including MirC, in *Aspergillus fumigatus.***

Eoin D. Mulvihill^1^, Nicola M. Moloney^1^, Rebecca A. Owens^1^, Stephen K. Dolan^1,2^, Lauren Russell ^1^ and Sean Doyle*^1^.

1. Department of Biology, Maynooth University, Maynooth, Co. Kildare, Ireland.

2. Department of Biochemistry, University of Cambridge, Cambridge CB2 1QW, UK.

***Corresponding Author**

Professor Sean Doyle

Department of Biology,

Maynooth University,

Maynooth,

Co. Kildare,

Ireland.

Tel: +353-1-7083858; Fax: +353-1-7083845; E-mail: [sean.doyle@nuim.ie](mailto:sean.doyle@nuim.ie)

Web: https://www.maynoothuniversity.ie/biology

**Table S1.** *A. fumigatus* strains used in this study.

| Strain | Reference |
| --- | --- |
| *A. fumigatus*ATCC46645 | http://www.atcc.org/ |
| Afs77 | http://www.fgsc.net/asperg.html |
| *A. fumigatus*∆*mirC*^ATCC46645^ | This study |
| *A. fumigatus*∆*mirC*^ATCC46645^(∆*mirC*^C1^) | This study |
| *A. fumigatus*∆*mirC*^ATCC46645^(∆*mirC*^C2^) | This study |
| *A. fumigatus*∆*mirC*^ATCC46645^(∆*mirC^GFP^*) | This study |
| *A. fumigatus*∆*mirC*^ATCC46645^(∆*mirC^GFP RFP^*) | This study |
| *A. fumigatus*∆*sit2*^Afs77^ | This study |
| *A. fumigatus*∆*ncr1*^Afs77^ | This study |
| *A. fumigatus*∆*impA*^Afs77^ | This study |
| *A. fumigatus*∆*impB*^Afs77^ | This study |
| *A. fumigatus*∆*impC*^ATCC46645^ | This study |
| *A. fumigatus*∆*impD*^Afs77^ | This study |
| *A. fumigatus*∆*impE*^Afs77^ | This study |

**Table S2.** Oligonucleotides used in this study.

| Primer name | Sequence (5’ to 3’) |
| --- | --- |
| *mirC*-P1 upstream F | CACAGCTCATCTTCGCTCAG |
| *mirC*-P2 upstream R AgeI | GAGAACCGGTAGGAGCGCGAGTGTAAATGT |
| *mirC*-P3 downstream F XhoI | GAGACTCGAGAGGGAGAAATCGAACCGAAT |
| *mirC*-P4 downstream R | TGTGGATTGTCGACCTGATG |
| *mirC*-P5 nested | AGCCCAAGAGGAGGACAAAT |
| *mirC*-P6 nested | GGAAGTCCGGTGGATGATAA |
| *sit2*-P1 upstream F | ATGTGATCAATGCCTCTGGA |
| *sit2*-P2 upstream R | GAGACAATTGCCTAGTAACCCGAGCGAAGG |
| *sit2*-P3 downstream F HindIII | GAGAAAGCTTGCACGCAGTGATCCGTTTAC |
| *sit2*-P4 downstream R | GGACAGTACTGCGGGAAGAG |
| *sit2*-P5 nested | TCAACTGCCCGCTCTTTTAG |
| *sit2*-P6 nested | TGCTCGAGAAACCAATGACA |
| *ncr1*-P1 upstream F | GGTGGTGTTTCTTTGCAGTG |
| *ncr1*-P2 upstream R AgeI | GAGAACCGGTTTCACCAGCTTCTTCCTCGT |
| *ncr1*-P3 downstream F XhoI | GAGACTCGAGCCATTCTGCAAATGACGATG |
| *ncr1*-P4 downstream R | ATCCACAACGATCATCACGA |
| *ncr1*-P5 nested | CGTCAGGCTGTCCAGGTTAT |
| *ncr1*-P6 nested | GGCGACAGAGCCTAAGATGA |
| *impA*-P1 upstream F | ATATCTTCTCGCCTCCATCG |
| *impA*-P2 upstream R AgeI | GAGAACCGGTAAGCGATACTGCGTCCATTT |
| *impA*-P3 downstream F XhoI | GAGACTCGAGTGAGCCTGCGGAATTAGTTT |
| *impA*-P4 downstream R | AATTTGTCTCCTCCCCCTTC |
| *impB*-P1 upstream F | GCGATTCCGGTTATTTGTCT |
| *impB*-P2 upstream R AgeI | GAGAACCGGTACCAAGGAACTGCCAAGATG |
| *impB*-P3 downstream F XhoI | GAGACTCGAGAGCTCCGTACTGGGTTTCAA |
| *impB*-P4 downstream R | AGATCGTGTAGCCGCACCT |
| *impB*-P5 nested | GTGGGTCGCTTCTTTGTTTC |
| *impB*-P6 nested | GATTTGCCTGCGCCTGTT |
| *impC*-P1 upstream F | ACACATCTGCTCCCCCATAC |
| *impC*-P2 upstream R SacI | GAGAGAGCTCGCTTCGCTGATGCTTTTACC |
| *impC*-P3 downstream F HindIII | GAGAAAGCTT AGCTACCGTGTCAACGTCCT |
| *impC*-P4 downstream R | GATCCATAATCGGGCTGCTA |
| *impD*-P1 upstream F | GTGGGGCTACCGCTGTATTA |
| *impD*-P2 upstream R AgeI | GAGAACCGGTCAGAAATGCCAGTGATTCCA |
| *impD*-P3 downstream F XhoI | GAGACTCGAGTAAGCTTGGGGACGAAGAGA |
| *impD*-P4 downstream R | AGAACCCGCTCAACAGACAC |
| *impD*-P5 nested | TGCGCTCTGTCATATTTTGC |
| *impD*-P6 nested | AGCATAGGACATGGCCAAAC |
| *impE*-P1 upstream F | GGAATGAGCGTCACACATGA |
| *impE*-P2 upstream R SacI | GAGAGAGCTCGTAACTGACGGCCAGGAAAA |
| *impE*-P3 downstream F HindIII | GAGAAAGCTTCGGCTCAGTGGATGGATATT |
| *impE*-P4 downstream R | GTCGAGATCGTGGGTGAACT |
| *impE*-P5 nested | AAGGCGGTGGGCTGTAATA |
| *impE*-P6 nested | CGTCATTGTCGGATTCACTG |
| PTR-optr-A1 | GAGGACCTGGACAAGTAC |
| PTR-optr-A2 | CATCGTGACCAGTGGTAC |
| *mirC*-RTPCR-F | CCCTTCTGTCCTTGCATCTT |
| *mirC*-RTPCR-R | GTAGCCTTGGACAACCTGGA |
| *sit2*-RTPCR-F | TCAAGGCTGAGAAGATGGGG |
| *sit2*-RTPCR-R | CTCTTGTATTCTGCGCGACC |
| *ncr1*-RTPCR-F | GCCTGAAGTATCGACGAGGA |
| *ncr1*-RTPCR-R | CTTTGCACGCAGGACATGAT |
| *impA*-RTPCR-F | GCTGCGGAATCTGACCTATG |
| *impA*-RTPCR-R | GACGAACACGGGGGACTG |
| *impB*-RTPCR-F | CTTGCGTCAAATGTCCTTCA |
| *impB*-RTPCR-R | AGACTAGCCAAGGCGCTGTA |
| *impC* -RTPCR-F | ACGGAGGTAAAAGCATCAGC |
| *impC* -RTPCR-R | GAAGCCAACGAGCAGGATAA |
| *impD*-RTPCR-F | AGCCTGTGTCGAAGAATTGG |
| *impD*-RTPCR-F | AGAAGCGAAAATCAGCCAAA |
| *impE*-RTPCR-F | TTGGTTGCTGGTGGTCTGTA |
| *impE*-RTPCR-R | AGAGGAACCGCAACAATCAG |
| *mirC*-comp-F-XhoI | GAGACTCGAGCACAGCTCATCTTCGCTCAG |
| *mirC*-comp-R-SacI | GAGAGAGCTCAGGCAATGATTTCGTTCACC |
| *mirC-*GFP-F-KpnI | ATAAGGTACC CCTTCTCTGTA |
| *mirC-*GFP-R-XmaI | TCTCCCCGGGCGTTCGTTTGACGTGTCTATT |
| *calm*-qPCR-F | CCGAGTACAAGGAAGCTTTCTC |
| *calm*-qPCR-R | GAATCATCTCGTCGACTTCGTCGTCAGT |
| *hapX*-qPCR-F | ATCCTAATTTCTCCCGGGCC |
| *hapX*-qPCR-R | CGTCGGTCAAAGTATCGCAA |

**Table S3.** Deleted regions in the knockout strains described in this paper.

| **Gene ID** | **No. of amino acids** | **Knocked out amino acids in deletion strain** | **Upstream sequence from ORF deleted (bp)** | **Downstream sequence from ORF deleted (bp)** |
| --- | --- | --- | --- | --- |
| *mirC* (AFUA_2G05730) | 611 | 1-598 | 93 | 0 |
| *sit2* (AFUA_7G04730) | 607 | 1-607 | 67 | 139 |
| *ncr1* (AFUA_6G09980) | 1273 | 76-756 | 0 | 0 |
| *impA* (AFUA_1G01690) | 512 | 34-504 | 0 | 0 |
| *impB* (AFUA_5G10510) | 1493 | 1-916 | 67 | 0 |
| *impC* (AFUA_3G03670) | 1314 | 18-1301 | 0 | 0 |
| *impD* (AFUA_5G07970) | 1540 | 1-931 | 89 | 0 |
| *impE* (AFUA_6G06620) | 174 | 1-174 | 49 | 84 |


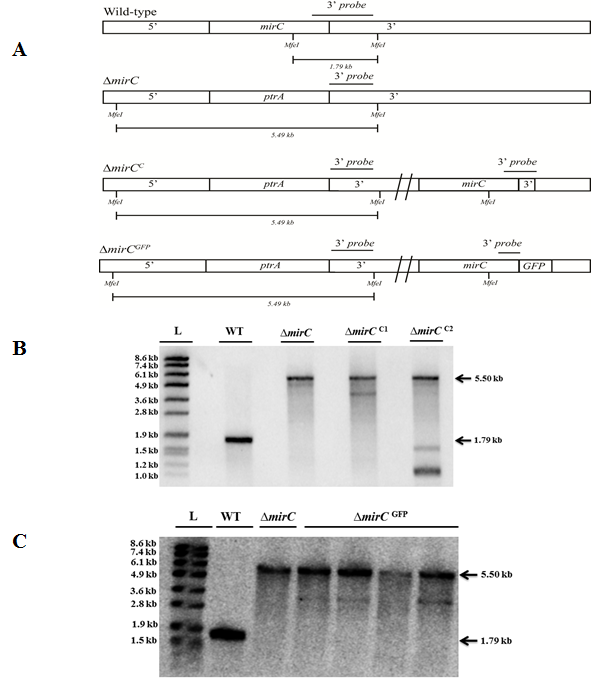


**Figure S1** (A) *A. fumigatus* *mirC* deletion and complementation strategy and schematic. gDNA was digested with *Mfe*I and a 3’ Digoxigenin (DIG) probe was generated using the primers *mirC*-P3 and *mirC*-P6. (B) Southern blot analysis of *mirC* deletion and complementation. Successful generation of a ∆*mirC* strain was confirmed with a band at 5.5 kb compared with the WT band of 1.79 kb as predicted. Generation of single copy complement (∆*mirC^C1^*) confirmed by additional band at ~4 kb. Generation of multi copy complement (∆*mirC^C2^*) confirmed by additional bands at ~1.5/0.9 kb. Estimation of complement sizes based on the DNA ladder fragments. (C) Southern blot of *mirC::GFP* fusion strain (∆*mirC^GFP^*). Four genomic extractions of the same ∆*mirC^GFP^* strain were analysed. Clear band at ~3 kb can be seen in the ∆*mirC^GFP^* strain. Restoration of *mirC* expression is also shown in Figure S2H, and MirC-GFP protein presence shown in Figure S8.


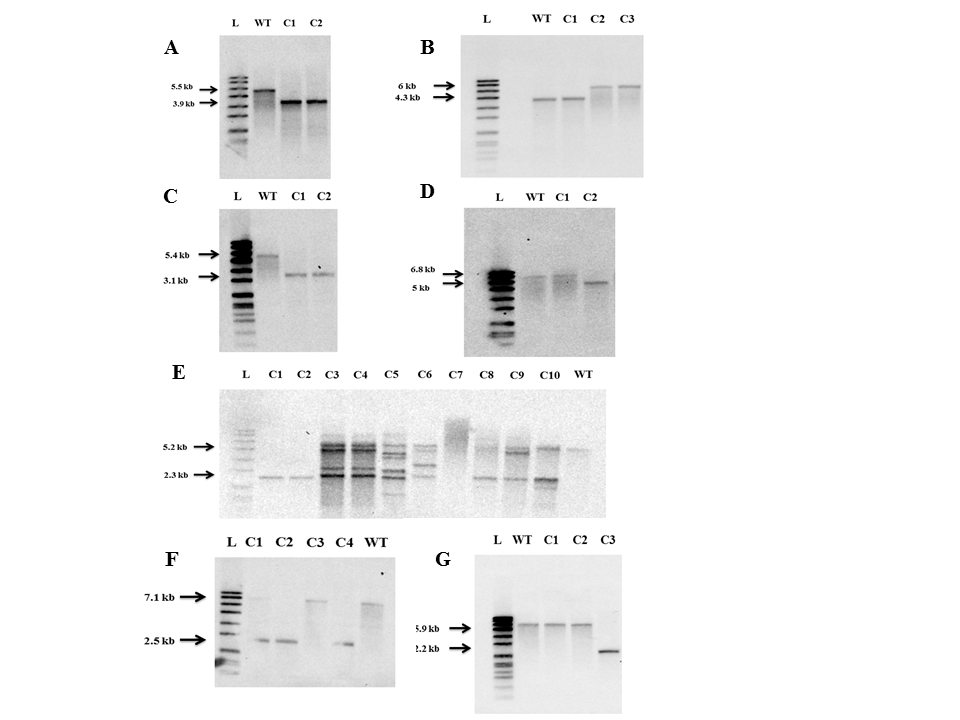


**H**

**
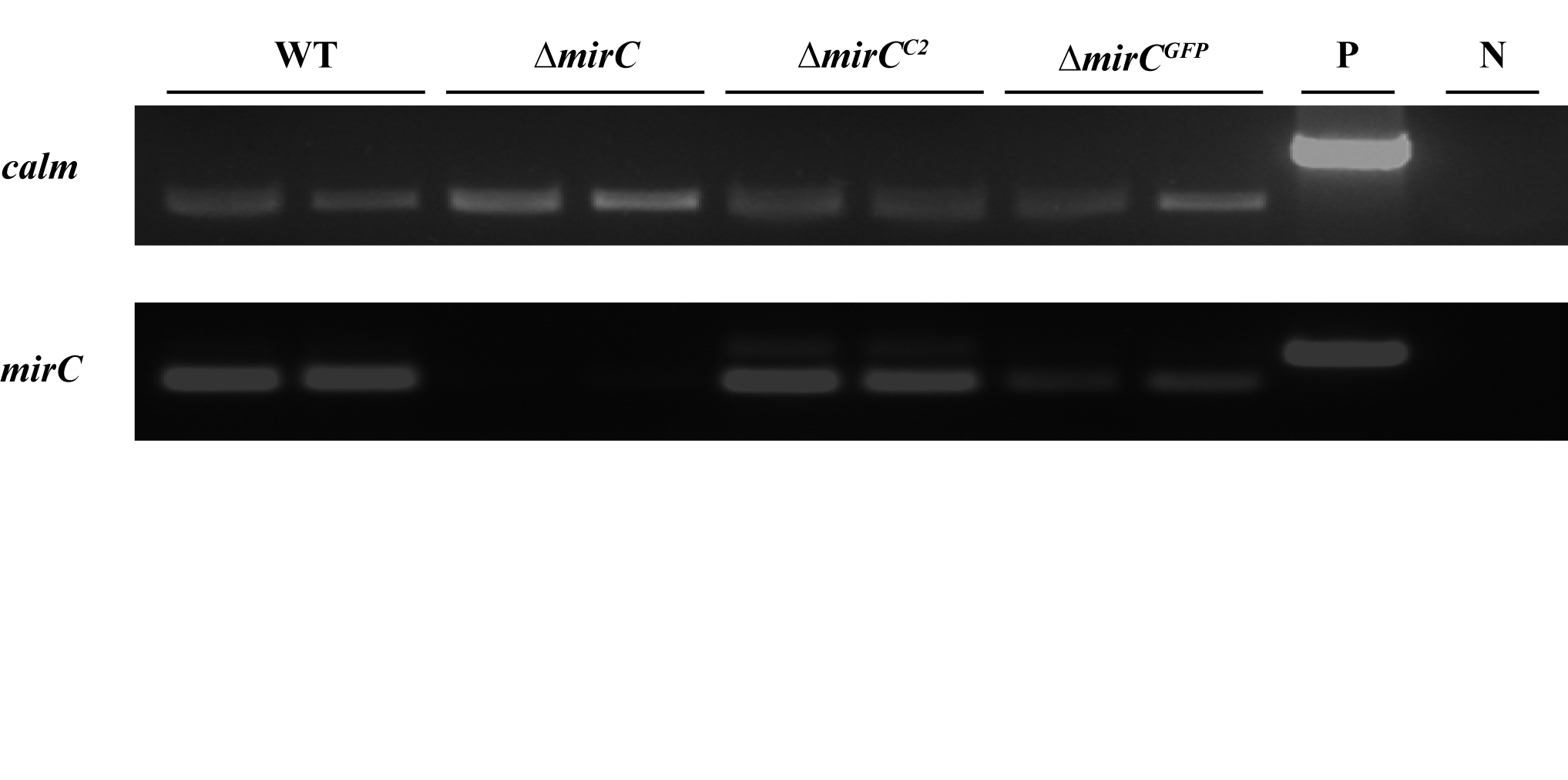
**

**Figure S2.** Southern blot analysis of (A) *sit2*, (B) *ncr1*, (C) *impA*, (D) *impB*, (E) *impC*, (F) *impD* and (G) *impE* gene deletions. (H) Compared to ATCC46645 (WT), confirmation of absence of *mirC* expression by RT-PCR (*n* = 2 biological replicates per condition) following growth in iron-deplete MM for 72 h at 37 ^o^C. Restoration of *mirC* expression in *∆mirC^C2^* and *∆mirC^GFP^* is also shown. Calm cDNA was used as a cDNA control (Burns et al., 2005). P represents amplification of genomic DNA while N represents a no DNA control PCR.

**Figure S3.** Phenotypic analysis of *A. fumigatus* ATCC46645 and ∆*impC*. The radial growth of *A. fumigatus* WT and ∆*impC* grown on MM plates under (A) iron sufficiency, (B) iron starvation (400 µM BPS), (C) iron excess (10 mM FeSO_4_), (D) 1 mM H_2_O_2_, and (E) 2 mM H_2_O_2_ after 48 h at 37 °C. (F) Dry weight (biomass) of *A. fumigatus* WT and ∆*impC* grown under iron starvation in MM. (G) RP-HPLC analysis of ferricrocin levels in dried mycelia from WT and ∆*impC* during growth under iron starvation (*n* = 3).

**Figure S4.** Phenotypic analysis of other *A. fumigatus* knockouts. The radial growth of *A. fumigatus* WT and knockout strains grown on MM plates under (A) iron sufficiency, (B) iron starvation (400 µM BPS), (C) iron excess (10 mM FeSO_4_), (D) 1 mM H_2_O_2_, and (E) 2 mM H_2_O_2_ after 48 h at 37 °C. (F) Dry weight (biomass) of *A. fumigatus* WT and knockout strains grown under iron starvation in MM.

**A.**

**B.**

**C.**

**Figure S5.** Detection of *A. fumigatus* siderophores. LC-MS/MS analysis of peaks collected following RP-HPLC analysis confirmed the identity of ferrated siderophores. (A) Extracted ion chromatogram (EIC) shows the elution of FSC^+Fe^, detected as a doubly charged ion by MS (M: 779.6, [M + 2H]^2+^: observed m/z 390.6; expected m/z 390.8). Fragmentation of the precursor ion is shown in the MS2 spectrum. (B) Extracted ion chomatogram (EIC), MS and fragmentation pattern (by MS2) of TAFC^+Fe^ (M: 905.8, [M + H]^+^: observed m/z 906.1; expected m/z 906.8). (C) Extracted ion chomatogram (EIC), MS and fragmentation pattern (by MS2) of FC^+Fe^ (M: 770.5, [M + H]^+^: observed m/z 771.0; expected m/z 771.6).

**Figure S6.** Siderophore production in *A. fumigatus* knockouts. RP-HPLC analysis of (A) TAFC^+Fe^ and (B) FSC^+Fe^ levels ­­­in the supernatants of *A. fumigatus* WT and knockout strains grown under iron-deplete conditions for 24, 48 and 72 h at 37 °C (*n* = 4). The supernatants were ferrated to a final concentration of 1.5 mM before the analysis. (C) RP-HPLC analysis of ferricrocin levels in dried mycelia from WT and knockout strains during iron-deplete growth (*n* = 3).

**Figure S7.** Comparison of RP-HPLC average peak areas (mAUs at 440 nm, *n* = 3) in the uptake of (A) TAFC^+Fe^ and (B) FSC^+Fe^ for WT, ∆*mirC* and ∆*mirC^C2^* strains. No statistical difference in the uptake of TAFC^+Fe^ and FSC^+Fe^ was observed between the different strains at the measured time-points. Statistical analyses were carried out using t-tests.

**Figure S8.** Immunodetection of intact MirC-GFP fusion protein in *A. fumigatus* *∆mirC^GFP^*. Whole cell lysates and microsomal proteins from ∆*mirC^GFP^* grown for 24 and 48 h under iron starvation were prepared as previously described (Moloney et al., 2016). Protein extracts were analysed by (A) SDS-PAGE and (B) Western blot probed with anti-GFP IgG, followed by anti-rabbit IgG HRP and developed with diaminobenzidine (DAB). Lanes show M: molecular-weight marker, 1: microsomal fraction at 24 h, 2: microsomal fraction at 48 h, 3: whole cell lysate at 24 h, and 4: whole cell lysate at 48 h. Bands for intact MirC-GFP (95 kDa) in whole cell lysates at 24 and 48 h were visible, in addition to lower molecular mass fragmentation products.
